# Cough detection using a non-contact microphone: A nocturnal cough study

**DOI:** 10.1371/journal.pone.0262240

**Published:** 2022-01-19

**Authors:** Marina Eni, Valeria Mordoh, Yaniv Zigel

**Affiliations:** Department of Biomedical Engineering, Ben-Gurion University of the Negev, Beer-Sheva, Israel; Valahia University of Targoviste: Universitatea Valahia din Targoviste, ROMANIA

## Abstract

An automatic non-contact cough detector designed especially for night audio recordings that can distinguish coughs from snores and other sounds is presented. Two different classifiers were implemented and tested: a Gaussian Mixture Model (GMM) and a Deep Neural Network (DNN). The detected coughs were analyzed and compared in different sleep stages and in terms of severity of Obstructive Sleep Apnea (OSA), along with age, Body Mass Index (BMI), and gender. The database was composed of nocturnal audio signals from 89 subjects recorded during a polysomnography study. The DNN-based system outperformed the GMM-based system, at 99.8% accuracy, with a sensitivity and specificity of 86.1% and 99.9%, respectively (Positive Predictive Value (PPV) of 78.4%). Cough events were significantly more frequent during wakefulness than in the sleep stages (*p* < 0.0001) and were significantly less frequent during deep sleep than in other sleep stages (*p* < 0.0001). A positive correlation was found between BMI and the number of nocturnal coughs (R = 0.232, *p* < 0.05), and between the number of nocturnal coughs and OSA severity in men (R = 0.278, *p* < 0.05). This non-contact cough detection system may thus be implemented to track the progression of respiratory illnesses and test reactions to different medications even at night when a contact sensor is uncomfortable or infeasible.

## Introduction

During sleep, different sounds can be recorded and analyzed. These sleep sounds can be related to physiological characteristics of the recorded individual, such as breathing, snoring, movement, speech, and coughing. Cough is a respiratory reflex considered to have defensive capabilities aimed at removing mucus and foreign particles from the lower airways [1]. It is also associated with many pathological conditions, most of which are related to the respiratory system (e.g., Chronic Obstructive Pulmonary Disease (COPD), asthma, and COVID-19), although others are seasonal such as allergies or colds.

**Fig 1:**
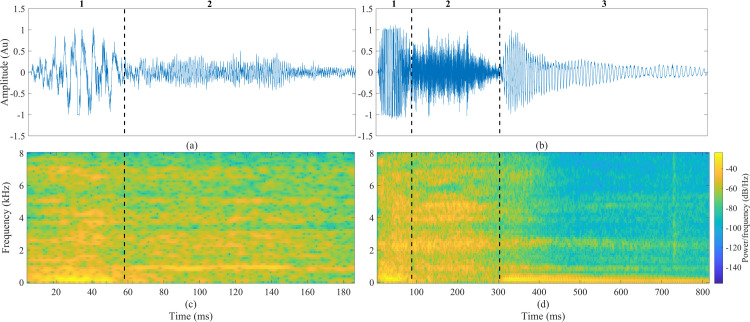
An example of two cough sounds–divided into its three phases: 1) explosive, 2) intermediate, and 3) voiced. (a) Cough event without the third phase in the time domain, (b) Cough event with a third phase. (c) Spectrogram of a cough event without the third phase. (d) Spectrogram of a cough event with the third phase.

A typical cough sound contains two or three phases. The first phase is an initial explosive phase with a very sharp increase in energy while air is released [2, 3]. The second phase is composed of a laminar airflow characterized by smaller amplitudes. The third phase (not always present) is composed of a turbulent airflow that includes a pitch frequency caused by activation of the vocal cords. An example of two recorded cough sounds with and without the third phase is shown in Fig 1, in the time and frequency domains.

Sleep is an essential function of the brain and plays an important role in an individual’s performance, learning ability, and physical movement [4]. Sleep is classically divided into five sleep stages: 1) wake, 2) Rapid Eye Movements (REM)–including rapid brain waves and eye movements accompanied by low muscle tone throughout the body, and three stages of Non-REM (NREM): 3) N_1_ or drowsiness–a transitional stage between wakefulness and stage N_2_, 4) N_2_ –a sleep stage that accounts for approximately 45%-55% of the sleep time (N_1_ and N_2_ are defined as light sleep), and 5) N_3_ –also known as slow-wave sleep or deep sleep [5].

Sleep disruption is common in patients who cough and is often why they seek medical attention [6]. The effects of cough on sleep and vice-versa are important and have been discussed in recent studies. It was reported that approximately 50% of all patients with chronic cough (cough for at least 8 weeks [7]) report sleep distruption from the cough [6]; chronic cough can severely affect daily life through fatigue, sleep disturbances and negative affect such as sadness [7]. Several works have investigated coughs during sleep [6, 8–10], and report that cough episodes are more frequent during wakefulness or during the daytime. However, little is known about the manifestation of coughs in different sleep stages. Power et al. [10] investigated the impact of cough on sleep in patients with Chronic Obstructive Pulmonary Disease (COPD). They analyzed the recordings of 10 patients using a directional microphone, and found that only one patient coughed during the REM stage and none during other sleep stages. These results on a small sample point to the need for more in-depth exploration based on a larger database of subjects and coughs, of all sleep stages.

Cough has recently been reported as a presenting symptom of Obstructive Sleep Apnea (OSA), which is a common sleep disorder affecting a large fraction of the population worldwide [11–14]. OSA is characterized by recurrent upper airway collapse during sleep that leads to inflammation, which can result in chronic unexplained cough [15]. The severity of OSA is usually defined by the Apnea-Hypopnea Index (AHI), which measures the number of apnea and hypopnea events per hour. The mean prevalence of OSA was reported to be 22% in men and 17% in women [16]. Daytime sleepiness is the main complaint among patients with OSA. Other common features include loud snoring, restless nocturnal sleep, and choking. Interventions for obstructive sleep apnea, such as Continuous Positive Airway Pressure (CPAP), have recently been shown to lessen the frequency of coughing [11–14]. It is important to know what sleep stages are involved during OSA events. Because REM sleep is associated with greater sympathetic activity, lower vagal tone, and more cardiovascular instability than non-REM sleep, obstructive events during REM sleep may disproportionately lead to hypertension and other adverse cardiovascular outcomes [17]. Since OSA is associated with coughs, much would be gained by investigating the prevalence of nocturnal coughs during different sleep stages.

Previous works have also explored nocturnal coughs and their association with different subject characteristics [18, 19]. One study analyzed nocturnal coughs and found a positive correlation between cough rate and age and BMI in Primary Ciliary Dyskinesia (PCD) patients [18], while [19] found that nocturnal cough was more frequent in asthmatic adults who were either overweight or obese.

The evaluation of cough intensity and its frequency of occurrence can provide valuable clinical information [20]. Currently, cough assessment is based on the administration of a patient-completed questionnaire [21] or the doctor’s opinion. These subjective measures tend to be biased [22]. By contrast, automatic cough detection, an objective measure of cough evaluation [9], can contribute to the diagnosis, the tracking of the progression of respiratory diseases, and testing for reactions to different medications, in addition to saving manpower.

There is growing interest in developing algorithms to automate the process of detecting cough events from audio signals. In Zigel et al. [9], whole-day (24h) audio recordings of 70 subjects equipped with wearable microphones were analyzed. In that study, the cough detection system was based on the adaptive Gaussian Mixture Model (GMM) and resulted in a sensitivity of 88.5%, a specificity of 95.6%, and a low Positive Predictive Value (PPV) of less than 20%. Another study [1] used the K-Nearest Neighbor (KNN) classifier with local Hu moments as inputs and achieved 88.51% sensitivity with a PPV of 87.51% and a specificity of 99.72%. You et al. [23] employed a Support Vector Machine (SVM) with non-Negative Matrix Factorization (NMF), and achieved an average sensitivity of 80.1%, a specificity of 83.1%, and a PPV of 83.5%. Matos et al. [24] used a Hidden Markov Model (HMM) with Mel-Frequency Cepstral Coefficients (MFCC) and achieved 71% sensitivity. Artificial Neural Networks (ANN) and Deep Neural Networks (DNN) have also been implemented in several studies to detect cough sounds using a variety of features; Drugman et al. [25] extracted 222 features including MFCC and its derivatives, spectral variation, and the spectral centroid. After feature selection, 50 features were fed into an ANN, resulting in classification performance of 89.85% sensitivity and 89.97% specificity (PPV not reported). Liu et al. [26], used a combination of a Hidden Markov Model (HMM) and DNN for classification with MFCC features as input and achieved a performance of 83.6% sensitivity and 90.9% specificity. A Convolutional Neural Network (CNN) was used in [27] on a database of 627 cough events, and reported a sensitivity of 86.8% and a specificity of 92.7%. Simou et al. [28] used mel-spectrograms and a Long-Short Term Models (LSTM) for cough detection on a database of 4,062 cough events. They achieved a sensitivity of 87.8%, a specificity of 98.9%, and an Area under the Curve (AUC) of 98.6% (PPV not reported). A comparative study was conducted by [29], which compared different sets of features and a variety of neural networks to classify cough events. The database was based on 3,114 cough events and 4,667 other sound events (such as speech). Their best system achieved an accuracy of 91.2% and an AUC of 0.965 (the sensitivity and PPV performances were not reported).

As suggested above, there are a number of main challenges to developing cough detection algorithms. 1) the variability of cough sounds between and within individuals, as well as the complexity of different respiratory diseases; 2) The differentiation of cough sounds from background noises or other sounds produced by the sleeper such as snoring, throat clearing, speech, sneezing, and other ambient sounds [24]. The former can lower the sensitivity of the algorithm for a particular recording, due to the lack of generalization of the algorithm to the type of cough sounds present in that recording, whereas the latter can lower its specificity and PPV, due to the occurrence of events incorrectly detected as cough events.

Here we present a reliable, simple automatic non-invasive cough detector that distinguishes spontaneous nocturnal coughs from snores and other sounds and noises (e.g., speech, manipulation of the device, drinking). The main contributions of this work are: (1) the usage of an extensive dataset containing nocturnal audio recording from 89 patients, (2) the development of dedicated features to best describe a variety of cough sounds, (3) the development of an innovative cough detection system based on DNN and a comparison to a GMM based system, (4) analysis of the correlations between nocturnal cough rate and sleep stages as well as between cough rate and OSA severity (AHI), age, gender, and Body Mass Index (BMI).

## Experimental setup

The data were collected at a university-affiliated Sleep-Wake Disorder center and biomedical signal processing laboratory. These routinely collected data were analyzed anonymously; therefore, informed consent was not required. The Institutional Review Committee of Soroka University Medical Center approved this study protocol (protocol number 10141). The institutional review board waived the need for written informed consent from the participants.

The database was composed of whole-night recordings (mean recording time 7.1 hours) of 89 adults with a variety of health conditions (asthma, bronchitis, sinusitis, etc.). All are typical to our region (Israel), do not sleep with CPAP, and represent a wide range of ages, BMIs (kg × m^-2^) and AHIs (events × hr^-1^) (Table 1). All the subjects were referred for a Polysomnography test (PSG), the gold standard for sleep stage evaluation (SomnoPro 19 PSG, Deymed Diagnostic, Hronov, Czech Republic) in the sleep-wake disorder unit (Soroka University Medical Center) for a sleep evaluation. PSG scoring includes sleep-wake patterns determined by a trained technician and underwent a second scoring by the head of the sleep lab. The scoring included labeling of each epoch (30 sec) as one of the 5 sleep stages using the PSG signals (for more details see [30]), and AHI was estimated by a trained technician. Audio signals were recorded from each participant by a digital audio recorder (Edirol, R4-pro, Shizuoka, Japan) connected to a condenser microphone (Rode, NTG-1, Sydney, Australia) that was suspended 1 m above the subject’s head. The audio signals were digitized at a sampling frequency of 44.1 kHz with 16-bit resolution and were synchronized with the PSG data.

**Table 1:** Subject characteristics.

Gender	BMI [kg/m^2^]	AHI	Age [y]	No. coughs
Male (62)	29.9±6.0	18.7±15.4	47.8±15.0	13.1±28.2
Female (27)	32.3±5.6	14.1±9.7	59.3±15.8	26.7±41.6
All subjects (89)	30.7±6.0	17.4±14.1	50.9±16.0	17.2±33.2

No. coughs stands for the total number of coughs in a recording. AHI stands for the average number of apnea and hypopnea events for each hour of sleep.

The dataset was randomly divided into three groups: train, development, and test (Table 2), while maintaining the balance of the number of events, AHI, gender age, and BMI in each dataset. A total of 1533 cough events were detected.

**Table 2:** Dataset split.

Dataset	Subjects	Division	No. coughs	No. snores	No. noises	Total events
Train	36	~40%	638	58,969	62,572	122,179
Development	27	~30%	435	44,884	49,470	94,789
Test	26	~30%	460	39,119	46,238	85,817
Total	89	-	1533	142,972	158,280	302,785

## Methods

**Fig 2:**
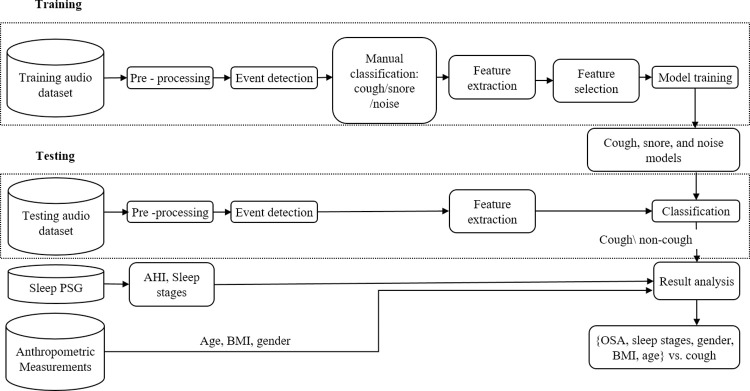
Block diagram of the cough detection system and analysis.

A cough detection system was developed to detect and analyze cough sound events in nocturnal audio files. Fig 2 presents a block diagram of the system. Since cough is a transient energetic sound event, detection of energetic events was performed; the sound events were further divided into three categories: cough, snore, and noise.

Each dataset group went through pre-processing, event detection, and feature extraction. All events in the training dataset were manually labeled and used to train the three system cough, snore, and noise models. The models were fed a selected set of acoustic features from the time and spectral domains.

This article has online Supporting Information, S1–S4 Datasets.

### Pre-processing

The pre-processing stage included DC removal and down-sampling from 44.1 kHz to 16 kHz since most of the relevant spectral information was below 8 kHz.

### Event detection

**Fig 3:**
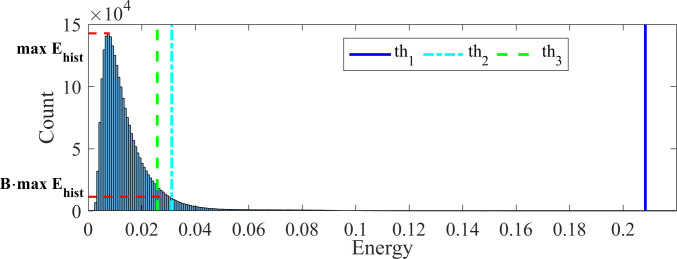
Energy histogram of a sample whole-night recording, with energy thresholds.

**Fig 4:**
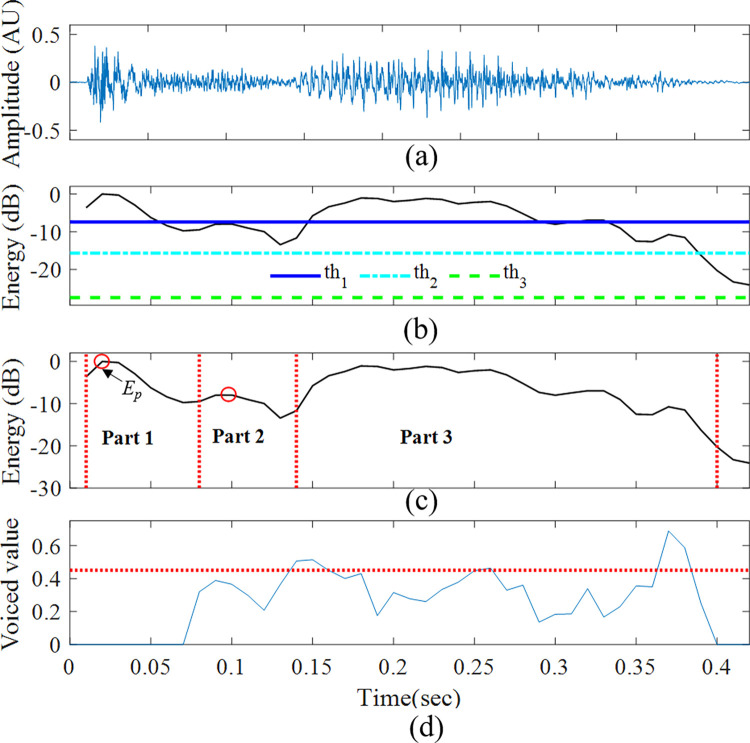
Detected cough event. (a) Signal in the time domain, (b) Energy of the detected cough event and the energy thresholds in dB, (c) Division of the event into three parts (dotted lines), (d) Voiced value over time. The voiced value was calculated for the detection of the 3^rd^ part using the autocorrelation peak of the two last parts (the detection threshold was set to 0.45). The open red circles in (c) mark the energy peaks of the first and the second parts (for the energy index and energy ratio features). The energy ratio feature was calculated as the ratio of the amplitude values of the two circles.

In the event detection step, to identify suspicious events as coughs, the energy was calculated from each frame (20ms length, 50% overlap) using the following equation:

$$E(j)=\overset{N}{\underset{i=1}{\sum }}x{\left(i\right)}^{2}$$ (1)

where *j* represents the frame number, *i* represents the sample index in the *j*^th^ frame, and *N* is the total number of samples in a frame. Energy and duration thresholds were applied to the energy signal. Three energy thresholds were calculated on each recording using the energy histogram calculated from the whole recording. The first energy threshold, *th*_1_, was used to detect the presence of a sound event and was calculated from a binned energy histogram, *E*_*hist*_, with the equation:

$$th_{1}=A\cdot E_{\mathrm{hist}}\{B\cdot \max\{E_{\mathrm{hist}}\}\}$$ (2)

where *E*_*hist*_{*j*} was the histogram value of the *j*^*th*^ bin. *B* was set to 0.08, and *A* was empirically set such that *E*{*B*∙max{*E*_*hist*_}}<*th*_1_<max{*E*}. The idea was to set an event detection threshold that was much higher than the background noise (the main histogram peak, see Fig 3). The purpose of the other two thresholds was to detect the beginning and end of each sound event (Fig 4B).

The beginning and the end thresholds were lower than *th*_*1*_ and were calculated by (3) and (4), respectively:

$$th_{2}=\max\{0.15\cdot th_{1},\arg\;\max\{E_{hist}\}\}$$ (3)

$$th_{3}=\max\{0.01\cdot th_{1},\;\arg\;\max\{E_{hist}\}\}$$ (4)

Thresholds *th*_*1*_*-th*_*3*_ are recording-dependent. Their values were calculated for each audio file (subject) using the energy histogram of that file and some global parameters; hence, the thresholds are not dependent on one specific recording setup. In addition, the constants (global parameters) that appear in Eqs ((2)–(4)) were chosen based on the training dataset, which enables repeatability of the method.

**Fig 5:**
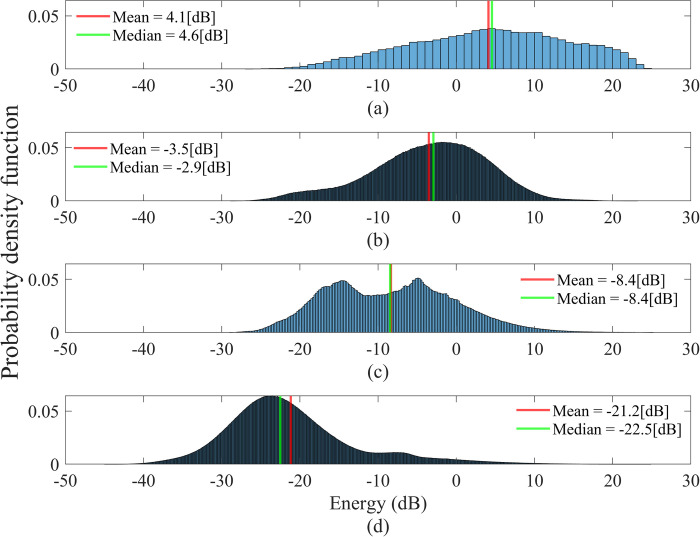
Energy distribution of different audio event types in the whole dataset. (a) The energy histogram of all cough events. (b) The energy histogram of all snore events. (c) The energy histogram of all noise events. (d) The energy histogram of all other events that did not pass the energy thresholds. The statistical analysis (Wilcoxon rank-sum test, α = 0.05) between (a)-(c) and (d) indicated a significant difference.

An example of an energy histogram of a whole night’s audio signal and its energy thresholds is shown in Fig 3. The energy histograms of each event type (cough, snore, noise), and the energy of other sounds (background noises), which did not pass the energy thresholds in the event detection are shown in Fig 5.

After detecting the events above the thresholds, the detection algorithm only kept those events with a duration between 0.15s and 1.1s (duration threshold), a common cough length [9].

### Manual classification

The manual classification (labeling) step was performed using a Graphical User Interface (GUI) by two listeners, with 93% agreement (Cohen’s kappa = 0.875), where most of the disagreements were between snores and noises. Each detected sound event from all the dataset groups was marked manually as a cough, snore, or noise sound. This was done to generate labels for the detected events, which were used later as the gold standard for classification and evaluation. For the labeled data information see S1 Table in the S1 Dataset.

### Feature extraction

A feature vector was extracted from each detected sound event. For precise values of the extracted features, see S2 – S4 Datasets in the supplementary information. These features were needed to characterize and differentiate cough sounds from other interference (such as snores and other noises). To extract these features, and since a cough sound has two to three parts (see Introduction), we automatically divided each detected sound event into two to three main parts. To define these cough parts, the energy peak, *E*_*p*_, was first calculated from the first part of the event (between 0 and 0.079s). The onset of the second part was defined as the first local energy minimum (*E*_*min1*_, see Fig 4C). A voiced feature (*Voiced*_*H*_−see description below) was calculated to determine the existence of a 3^rd^ part of the event; this feature was calculated from each frame in the event, starting from the onset of the second part (see Fig 4D). The onset of the third part, if present, was determined when *Voiced*_*H*_ > 0.45 (*E*_*min2*_, Fig 4C and 4D). Based on this feature, the proportion of the voiced versus unvoiced coughs was 2:3, respectively. From each detected sound event, 34 features were extracted from 12 feature types (Table 3) as detailed below.

**Table 3:** Features and the selected feature subsets.

Feature type	Number of features	Selected Features
Energy index	1	1
MFCC 1^st^ part	12	11
MFCC 2^nd^ part	12	12
*Voiced*_H_	1	1
*Voiced*_L_	1	1
Variance of LPC	1	1
Energy ratio	1	1
ZCR	1	1
Kurtosis	1	0
Skewness	1	0
Entropy	1	1
Spectral centroid	1	1
**Total**	**34**	**31**

#### Energy index

The frame (time) index of maximum energy in the first part of cough sound (Fig 4C).

#### Mel Frequency Cepstral Coefficients (MFCC)

A widely used set of spectral features in speech processing [29]. Twenty-four cepstral features were obtained: 12 MFCCs from the first part of the event and 12 from the second part.

#### Voiced_H_

Since some coughs have a voiced part (the third part), and since the fundamental frequency of the vocal cords is usually between 50 and 255Hz [31], a voiced feature was determined. This feature was calculated as the amplitude of the autocorrelation peak in the region of these frequencies [32]. This feature contributed to discriminating between voiced coughs and non-voiced noises.

#### Voiced_L_

The assumption was that snores are usually more harmonic (semi-periodic) in this low range of frequencies (10-100Hz) [33]. For this reason, this feature was used to better separate coughs from snores.

#### LPC variance

A measure of stationarity using spectral content. From each frame, eight LPCs were extracted and the variance of each coefficient was calculated. The LPC variance feature is defined as follows:

$$LPC_{\sigma^{2}}=\frac{1}{M}\overset{M}{\underset{m=1}{\sum }}\frac{1}{K-1}\overset{K}{\underset{k=1}{\sum }}{\left(a_{m}(k)-{\bar{a}}_{m}\right)}^{2}$$ (5)

where *K* is the number of frames of the detected event, ${\bar{a}}_{m}$ is the mean value of the *m*^th^ LPC along the event, *M* is the number of LPCs, and *σ*^2^ is the notation for variance.

#### Energy ratio

Given the exploding phase of the first part of a cough [34], the assumption was that the ratio between the energy peaks of the first part and second part of a cough event would usually have higher values than other sounds (Fig 4C).

#### Zero-Crossing Rate (ZCR)

The mean ZCR value was calculated from all frames in the detected event. It usually results in higher values for noisy and high-frequency signals where sign changes are more frequent [35].

#### Kurtosis

The kurtosis, which measures how heavily the tails of a probability distribution of the sound differ from the tails of a normal distribution, was calculated on the audio sample values of the detected sound event.

#### Skewness

The skewness, which measures the asymmetry of the probability distribution of the sound, was calculated on all the sample values of the detected sound event.

#### Entropy

Entropy was defined as the average rate at which information is produced by a stochastic source of data. Here, the entropy was estimated on the time samples of the detected event, as in:

$$H=-\sum_{i=1}^{n}P(x(i))\cdot \log_{2}(P(x(i)))$$ (6)

where *P* is the appearance probability of *x(i)*.

#### Spectral Centroid (SC)

A measure that indicates where the "center of mass" of the spectrum is located. This feature was calculated with the general equation:

$$SC=\frac{\sum_{i=1}^{n}f(i)X(i)}{\sum_{i=1}^{n}X(i)}$$ (7)

where *f(i)* is the frequency of the *i*^th^ spectral bin and *X* is the Fourier transform of *x*.

### Feature selection

**Fig 6:**
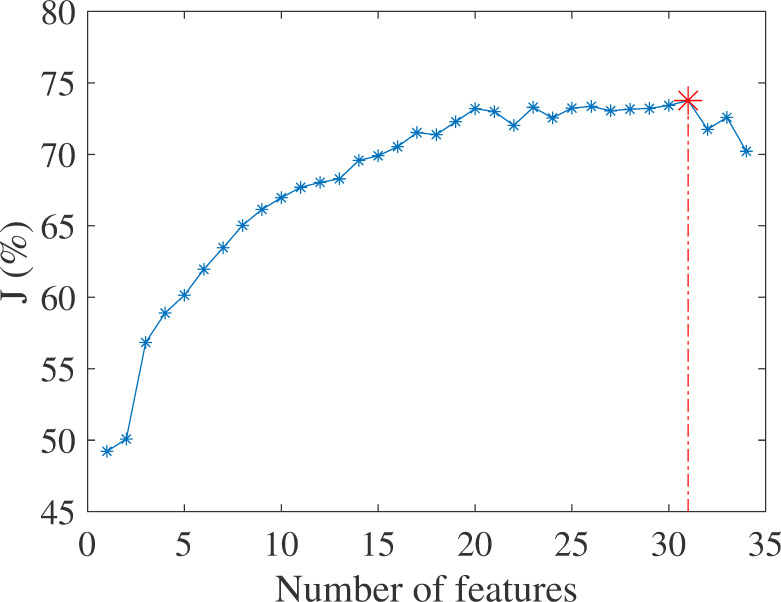
Feature selection criterion value vs. the number of selected features for the GMM-based system.

Feature selection is used to find a subset of features that are the most relevant to the data. The algorithm here was based on Sequential Forward Selection (SFS) [36] and a criterion, *J*, which was calculated using the calculated sensitivity and PPV of the GMM classifier:

$$J=\frac{1}{2}\cdot \mathrm{Sensitivity}+\frac{1}{2}\cdot PPV$$ (8)

where the definitions of Sensitivity and PPV appear in the Evaluation measures section. For this purpose, the feature selection algorithm used the training and the development datasets and their true labels. This method saves the most informative features at each step by maximizing the criterion and choosing the next feature depending on features that have already been selected. The selected features for each step were stored with their criterion values. The feature subset that achieved the highest criterion value was then chosen (see in Fig 6).

### Classifiers

Two different classifiers were developed and tested: a GMM and a DNN.

#### GMM

*Training*. To determine the GMM order; i.e., the number of Gaussians per model (cough, snore, and noise), multiple combinations of Gaussian numbers were tested (up to 20 Gaussians per model). For feature selection, the models were trained on the training dataset and were evaluated on the development dataset to calculate the feature selection criterion (8). The selected feature subset is shown in Table *3* and Fig 6). The test dataset was only used for the final analysis (see Results section).

*Classification (testing)*. This step used the extracted set of features from the detected events and three trained GMM models to classify the test dataset. To determine whether the event was a cough, each model produced a likelihood score for belonging to this model (9):

Sj=p(β|λj)=∑i=1mj(ωi⋅1(2π)D2|∑|12⋅exp{−12(β−μi)TΣi−1(β−μi)}) (9)

where **β** is the feature vector; *λ*_*j*_ is the *j*^*t*h^ model (*j* = 1,2,3): cough, snore, or noise; *m*_*j*_ is the number of Gaussians in the *j*^th^ model; *ω*_*i*_ is the weight of each Gaussian in the mixture model; **μ**_*i*_ is the mean of the *i*^th^ component of the model; **Σ**_*i*_ is the covariance matrix of the *i*^th^ component of the model, and *D* is the size of the feature vector **β**.

After calculating the three probabilities for each event, a Log-Likelihood Ratio (LLR) score was calculated. In order to differentiate between coughs and other sound events, the probability of belonging to the cough class (*S*_*1*_) was divided by the maximum probability of being included in the snore (*S*_*2*_) or noise class (*S*_*3*_), as described in the following equation:

$$LLR=\log(\frac{S_{1}}{\max(S_{2},S_{3})})$$ (10)

**Fig 7:**
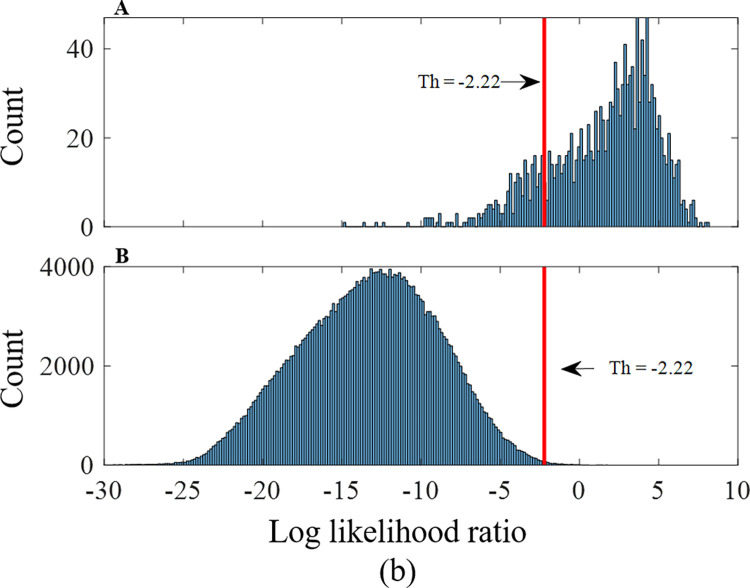
Histograms of LLR scores with the LLR threshold (DNN system, whole dataset). (a) Distribution of LLR scores for coughs (1533 events). (b) Distribution of LLR scores for snores and noises (301,252 events).

The threshold was set according to the Receiver Operating Characteristic (ROC) curve of sensitivity vs. the PPV for different thresholds, as shown in Fig 7. Above this threshold, the events were marked as coughs.

#### DNN

**Fig 8:**
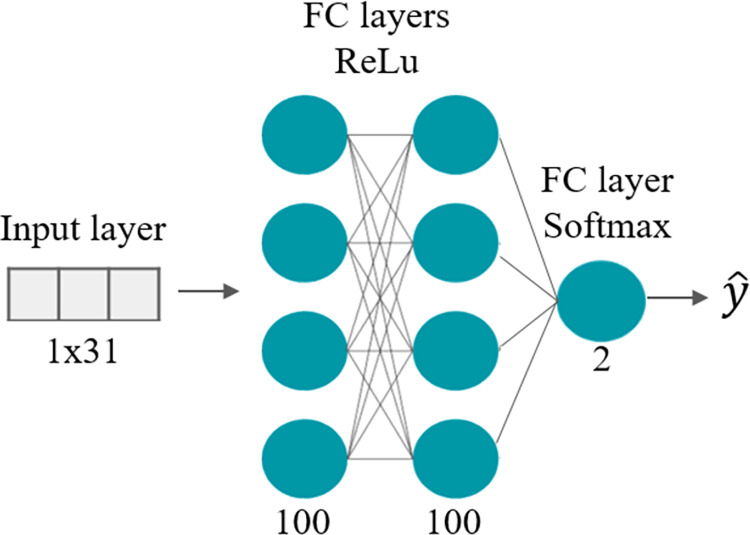
Neural network architecture. The network gets a feature vector for each event as input from a test dataset group and derives a binary output for 2 classes (cough and non-cough) using a Softmax activation function. Both FC hidden layers had 100 neurons and worked with the ReLu activation function.

*Training*. The model architecture is presented in Fig 8. The model’s input was the training dataset containing the same selected features as the previous classifier. Several model parameters and architectures were evaluated for classification. The chosen model was trained using an Adam optimizer (an extension to stochastic gradient descent), with a learning rate of 5×10^−6^ and a batch size of 32. The model was a simple DNN, which contained two Fully Connected hidden layers with a 50% dropout in between. The model had 13,502 learnable parameters and the training converged after 250 epochs. To deal with the unbalanced data, class weights were integrated into the model. Although a GMM model was trained with a three-class classification, the best classification results for the DNN were obtained with a model trained on two classes, where the snore and noise events were merged into one class.

*Classification (testing)*. Each detected sound event from the test dataset underwent feature extraction; this feature vector was the input to the DNN classification system. The model outputted two probability scores (by the softmax output layer), one for each class (*S*_1_ –cough, *S*_2_ –non-cough). Then, the LLR score was calculated by:

$$LLR=\log(\frac{S_{1}}{S_{2}})$$ (11)

Events whose LLR exceeded a pre-defined threshold (see Results section) were marked as coughs.

### Evaluation measures

The evaluation parameters described below were implemented.

#### Accuracy

The percentage of correct classifications out of the total number of examined events.

#### Sensitivity (recall)

The percentage of coughs that were correctly identified out of the total number of true-labeled coughs.

#### PPV (precision)

The percentage of coughs that were correctly identified out of the events that were classified as coughs.

#### Specificity

The percentage of non-cough events that were correctly classified.

#### NPV (negative predictive value)

The percentage of coughs that were correctly identified as non-coughs out of the total number of true-labeled non-coughs.

#### Cohen’s kappa (κ)

This measure is very useful for multiclass problems. Specifically, it takes the unbalanced deviations of the different classes into account [37].

### Cough analysis

After cough detection using the best classifier (DNN in this case, see Results), the detected cough events were analyzed (cough rate, #/hour) as a function of the subjects’ characteristics; namely, AHI, BMI, gender, age, and sleep stages. A Wilcoxon rank-sum test (*α* = 0.05) [38] was used to assess the significance of the results.

## Results

### Cough detection

The cough detection algorithm was trained on *36* subjects and tested on 26 subjects (Table 1). The feature selection algorithm yielded a set of 31 features (see Fig 6 and Table *3*). These features were then used for training and testing both classifiers.

#### GMM

**Fig 9:**
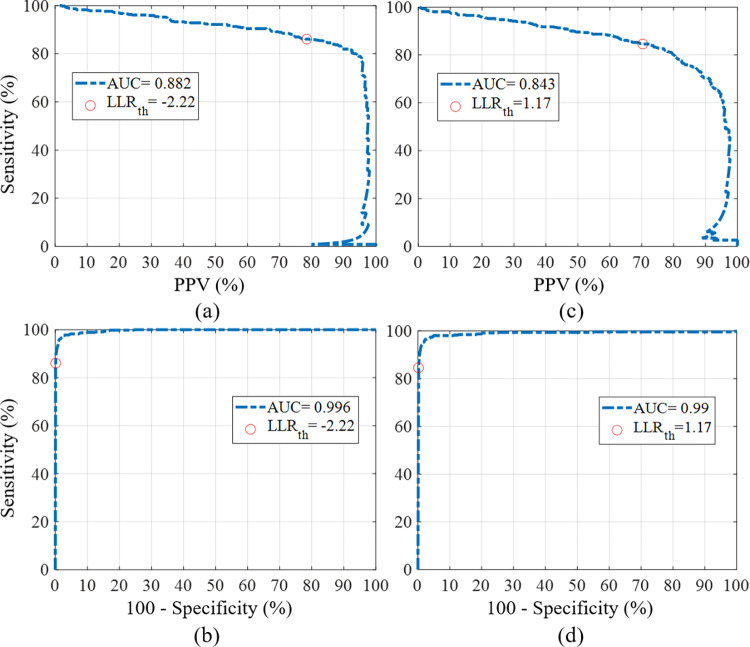
ROC plots of the cough detection system (test dataset). (a), (c) Sensitivity vs. specificity of the DNN and GMM systems, respectively. (b), (d) Sensitivity vs. PPV of the DNN and GMM systems, respectively. The open red circles mark the value at the LLR thresholds.

Different model orders were tested using the training and development datasets; the optimal model orders assigned were one Gaussian for the cough model, one Gaussian for the snore model, and 16 Gaussians for the noise model. The LLR score was calculated from each detected sound event. The distribution of LLR values for each of the classes can be seen in Fig 7. These distributions exhibited a good separation between coughs and non-coughs. The sensitivity, specificity, and PPV were calculated by comparing the LLR scores of each event to an LLR decision threshold. Analyzing the Area Under Curve (AUC) of the ROC curve for the two classifiers on the *development* dataset yielded an AUC of 0.984 for GMM and 0.995 for DNN. The precision-recall-AUC was higher in the DNN (0.86) than the GMM (0.794). Therefore, the DNN was chosen as the classifier for further analysis of coughs during sleep. Fig 9 depicts two ROC plots of these performance measures on the *test* dataset. To find a suitable LLR decision threshold for classification, the performance measurements were examined on the development dataset: an arbitrary LLR threshold was set to 1.17 to achieve a reasonable compromise between sensitivity and specificity (sensitivity of 85.06%, and a specificity of 99.64% for the development dataset).

#### DNN

In addition to the GMM classifier, a DNN classifier was tested as well. The LLR decision threshold in this system was set to be -2.22 to maintain sensitivity of 85.06% as in the GMM classifier. Tables 4 and 5 show the confusion matrices of the test dataset classification for the GMM and DNN models, respectively, in classifying coughs versus non-coughs. Table 4 shows the results after the fusion of the classified noise and snore events.

**Table 4:** Confusion matrix for the GMM classifier (test dataset).

		True class	
		Cough	Non-cough
**Predicted class**	**Cough**	84.57%	0.19%
	**Non-cough**	15.43%	99.81%

**Table 5:** Confusion matrix for the DNN classifier (test dataset).

		True class	
		Cough	Non-cough
**Predicted class**	**Cough**	86.09%	0.13%
	**Non-cough**	13.91%	99.87%

Since the data were unbalanced (fewer coughs than non-cough sounds), Cohen’s kappa was calculated as well. The tables show that the classification results of the DNN outperformed the GMM; for similar sensitivity values, Cohen’s kappa values were 0.77 and 0.82 for the GMM and the DNN, respectively.

To assess the generalization of the classifiers, a 5-fold cross-validation was conducted for each classifier (see Table 6). It compares a GMM classifier that was trained to differentiate between coughs, snores and noises (multiclass classification) to a DNN classifier that was trained to differentiate between coughs and non-coughs (binary classification). The results reported in this table were obtained after the fusion of the classes in the test phase of the GMM classifier.

**Table 6:** Classification results for the 5-fold cross-validation.

Classifier	Accuracy [%]	Sensitivity [%]	Specificity [%]	PPV [%]
GMM	99.37 ± 0.516	85.07 ± 0.05	99.44 ± 0.51	51.07 ± 20.78
DNN	99.39 ± 0.53	85.52 ± 2.61	99.46 ± 0.52	57.43 ± 30.75

### Analysis of detected coughs during sleep

Based on the participants’ PSG results, all the detected coughs (from all datasets) were assigned to their wake/sleep stage (wake/REM/N_1_/N_2_/N_3_, see S2 Table in S1 Dataset) and the number of coughs was normalized according to the sleep stage duration of each subject (cough rate). S3 Table in the S1 Dataset details the duration of each sleep stage for each subject. S4 Table in the S1 Dataset presents the resulted normalized values for each subject in each corresponding sleep stage. Each subject’s audio file was analyzed between lights off and lights on (the time annotation of lights off and on in the subject’s room).

**Fig 10:**
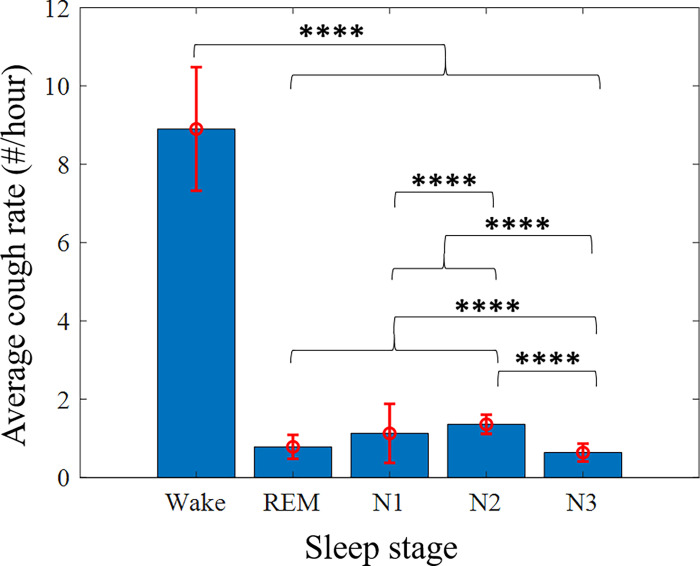
Average cough rate vs. wake/sleep stages. The average cough rate was calculated as the number of coughs per subject, normalized by the duration of each wake/sleep stage. The red lines are the standard errors. ****p ≤ 0.0001.

Fig 10 shows the average cough rate for each wake/sleep stage. It indicates that most of the coughs detected during the night occurred in the wake phase and fewer during the sleep stages (*p* < 0.0001). In addition, there was a significant difference (*p* < 0.0001) between deep sleep (N_3_) and the other sleep stages (N_1_, N_2_, & REM) where there was a considerably smaller number of coughs in the deep sleep stage. There was also a significant difference between deep sleep (N_3_) and light sleep (N_1_ & N_2_) (*p* < 0.0001), and between the drowsiness stage (N_1_) and the most frequent stage (N_2_) (*p* < 0.0001).

**Fig 11:**
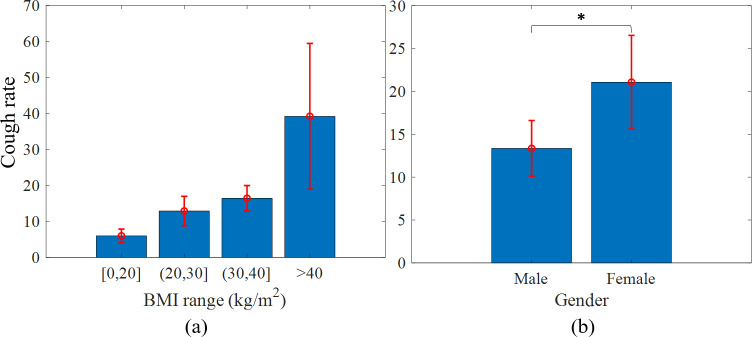
BMI and gender vs. cough rate. (a) Cough rate in different BMI ranges. (b) Cough rate of males and females. *p < 0.05.

**Fig 12:**
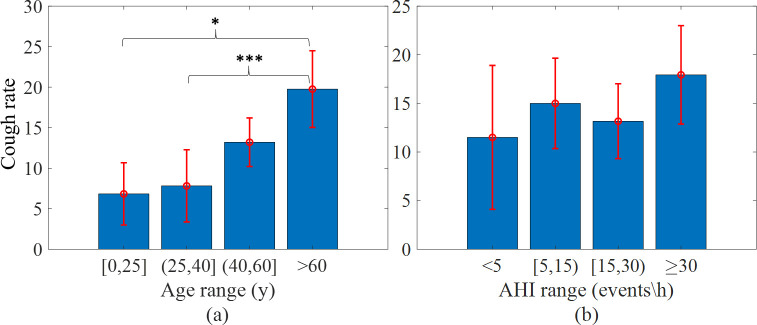
Age and AHI values vs. cough rate. (a) Cough rate in subjects above and below age 50. *p <0.05, **p<0.01, ***p<0.001. (b) Cough rate in different AHI ranges.

The correlations between the subjects’ number of coughs (cough rate, cough frequency) and their demographics, including gender, age, AHI, and BMI are depicted as histograms in Figs 11 and 12. The distribution of coughs for BMI shows that most of the coughs occurred in subjects with higher BMIs (Pearson R = 0.232, *p* < 0.05). A separate analysis for women and men revealed that women with higher BMIs (obese) had more nocturnal coughs than women with lower BMI values (Pearson R = 0.414, *p* < 0.05). However, when removing age, the partial correlation between the number of coughs and BMI was not significant, probably because there is a correlation between BMI and age (Pearson R = 0.237, *p* < 0.05). In general, women coughed significantly more than men (*p* < 0.05), but there was a similar number of coughs in each AHI range with relatively wide standard errors (Fig 12B). There was a weak positive correlation (Spearman R = 0.278, *p* < 0.05) between the AHI and the number of coughs in men (no correlation for women).

Notably, age emerged as an important factor. Fig 12A shows that most of the coughs occurred in older subjects (Spearman R = 0.387, *p* < 0.001). A partial correlation, excluding BMI and gender, also indicated a significant result, with R > 0.3. Furthermore, there were significantly more coughs in subjects older the age of 40 (*p* < 0.01) compared to the younger subjects (age thresholds of 50y and 60y were similar, *p* < 0.001 and *p* < 0.01, respectively). There was also a significant difference between the cough rate of subjects under the age of 25 versus subjects above 60 (*p* < 0.05), as well as between subjects aged 25–40 and subjects over the age of 60 (*p* < 0.001).

## Discussion

### Cough detection

In this paper, an algorithm for automatic cough sound detection using a non-contact microphone was presented. The detection system was specially designed to detect and analyze nocturnal audio recordings, by taking nocturnal sound events such as snoring, breathing, and choking into account. The cough sound events were detected from whole-night recordings of 89 subjects referred to the sleep laboratory. After sound event detection, a 34-dimension feature vector was extracted from each detected sound event. These features were defined to provide a good acoustic separation between coughs and other nocturnal sound events. Analysis of the skewness and kurtosis values of coughs versus non-cough events confirmed a significant difference between the two (*p*<0.0001). Feature selection was used to reduce feature space dimensionality. Two types of classifiers were implemented for event classification: GMM and DNN. For comparison purposes, we fed the two classifiers the same feature set.

Compared to other studies in the field of cough detection [1, 9, 27, 29] our DNN model obtained the highest accuracy and specificity values (above 99.8%). It also had a higher PPV than most studies, as well as a high sensitivity rate. However, each system was evaluated on a different database, different environments, and equipment. The DNN classifier demonstrated better performance with an accuracy of 99.8% (86.1% sensitivity, 99.8% specificity, and 78.4% PPV). The GMM produced similar accuracy and sensitivity (99.7% and 84.6% respectively); however, the PPV was much lower (70.3%). Note that it is possible to raise the sensitivity to above 90% using different LLR thresholds, but at the expense of the PPV, which adds more false alarms of detected coughs (e.g., a LLR_th_ of -2.8 will result in a sensitivity of 90% and PPV of 67.1% using the DNN classifier). In addition, we also examined a GMM classifier that was trained on two classes, and a DNN classifier that was trained on three classes; however, their performances were inferior.

We split the data into train, development (validation), and test using different subjects (patients), so there were no common subjects between the three datasets. The model parameters and the decision threshold were determined using the development dataset, and the test dataset was reserved for the final evaluation. In addition, we evaluated a 5-fold cross-validation to observe the generalization of the system. Similar accuracy, sensitivity, and specificity values were achieved, whereas the PPV value was somewhat lower. The key advantage of this system is that it is relatively simple and fast. Although it is a DNN-based system, it can run on a PC without GPU: a processing time (during the test) of ~1min is required for each hour of audio recording (processor: Intel i7-7700 CPU@3.60GHz).

For comparison, in a pilot study, we implemented a CNN-based cough detection system. We used mel-spectrograms (128x109) for each sound event as input to the CNN model. The network was composed of five convolutional layers, three max-pooling layers, two batch normalization layers, and three dense layers. This network totaled 87,795 learnable parameters, which is approximately 6 times more than our DNN model, and required 12 fold additional processing time. The preliminary results on the test dataset showed better PPV values (94.7%), but lower sensitivity values (81.5%). In future work, we plan to further develop and test this system.

### Analysis of detected coughs during sleep

The results of the analysis of the clinical data were performed on both the test and all subjects. However, in order to draw more accurate conclusions and for statistical robustness, we chose to present the analysis on the complete large database.

One of the main novelties of this work is the focus on nocturnal audio signals to better understand the manifestation of coughs between lights off and lights on. Correlations were calculated between sleep stages and the relative cough rate. The findings indicated that coughs occurred significantly less often during the sleep stages and more frequently during the wake stage. These results are consistent with previous studies [6, 8–10] that reported similar findings; for example [6, 9] analyzed 24-hour recordings and showed that coughs were more frequent during the daytime. Furthermore, our analysis showed that most of the coughs that occurred during sleep were less frequent in deep sleep (N_3_) than in the other sleep stages (REM, N_1_, and N_2_).

When analyzing the transition between sleep stages when cough occurs, we found that when cough occurred during N2, in 10% of the times the next stage was awake, and in the rest of the time it remained in N2. When cough occurred during N3, in 6% of the times the next stage was N2 (the rest remained in N3). When cough occurred during REM, there was no transition to other sleep stages, except wake (3% of the time). We also examined the cases where cough caused awakenings. We found that in 92.1% of the time, the subject was already awake, in 7.7% of the time the cough occurred during N2, and in 0.2% of the time the cough occurred during REM. Please note, that the sleep stages were annotated for 30-second epochs.

Further statistical analysis of the subjects’ demographics indicated that higher BMI values were related to a higher cough rate (Fig 11). Similar results were reported in [18], where a positive correlation was found between the nocturnal cough rate and BMI in Primary Ciliary Dyskinesia (PCD) patients. In general, [7] indicated that the chronic cough risk is twofold to threefold higher in obese individuals in the general population. This may be attributed to excess fat mass in the airways that causes cough production [39]. A significant positive correlation between age and the number of nocturnal coughs emerged here (Fig 12A), as was also found in [40]. Finally, the comparison of the cough rate in terms of gender revealed that women coughed significantly more than men during the night, as was also found in [40] on a database of 100 subjects (65 women), and in [41] on a database of 933 subjects (52.2% women). One possible explanation for this outcome is that women have more episodes of upper airway resistance, which may cause cough episodes [42].

According to [14, 43], there is an association between cough and OSA; specifically [43] suggested that upper and lower inflammation and trauma to the upper airways during each apnea-hypo-apnea episode observed in OSA, may contribute to chronic cough. Several studies have investigated the prevalence of OSA in patients with chronic cough; [11] found that 44% of 75 subjects were found to have OSA.

There was no significant correlation between OSA severity and the number of nocturnal coughs in terms of the severity of OSA in women, but there was a weak positive correlation with the severity of OSA in men. This finding may be attributed to the fact that OSA is more prevalent in men [42] and that in our database the AHI mean and variance were higher in men (18.70 ± 15.43 vs. 14.12 ± 9.68 in women). When removing the smokers (16) from the entire database, a weak positive correlation emerged (R = 0.304, *p* = 0.05). These findings are somewhat similar to [44], which was based on a database of non-smokers. They found that OSA patients (AHI = 53.6 ± 24.7\h of sleep) had a significantly higher incidence of chronic cough than the controls (no OSA).

Our database included subjects with a variety of health conditions who were referred to a polysomnography test. Future research should test larger samples to introduce more variety and draw more pinpointed conclusions.

## Conclusion

This article presented a reliable DNN-based automatic nocturnal cough detector using a non-contact microphone. Thirty-one selected features in the time and frequency domains were used to characterize the cough patterns. The classification results confirm that the algorithm can clearly distinguish between coughs and other noises in a sleep environment. The main findings indicated that: 1) cough events were significantly more frequent during wakefulness than during sleep, 2) in deep sleep (N_3_) the cough rate was significantly lower than the other sleep stages, 3) the number of nocturnal coughs was significantly higher for women, 4) the number of coughs was positively correlated with age and BMI, and 5) the number of coughs in men was positively correlated with OSA severity.

Future work could implement this system to better track the progression of respiratory illnesses, and to test reactions to different medications, especially in these times of a world pandemic.

## Supporting information

S1 Dataset S1 Table: Subjects characteristics and number of manually labeled events. This table lists AHI, gender, and BMI, where the values 1 and 2 in the gender column indicate men and women, respectively. It provides the total number of events (cough, noise, snore, and coughs between the lights off/on separately) detected using manual labeling. NA stands for Not Available. S2 Table: Number of detected coughs per participant and stage. This table lists the number of coughs detected using the DNN model for each subject, according to each sleep stage and the total number of coughs detected for each subject. S3 Table: Stage duration per patient [hours]. This table lists the duration of each sleep stage, in hours, for each subject detected during the PSG test. S4 Table: Normalized number of detected coughs per participant and stage. This table shows the values of S2 Table normalized by the values in S3 Table. These data support the results of Table 1, Figs 10–12.
(XLSX)

S2 Dataset S1 Table cough: Extracted features of each manually labeled cough event. The table also contains the subject ID for each event, and its associated dataset (train, validation\valid, or test).
(XLSX)

S3 Dataset S1 Table snore: Extracted features of each manually labeled snore event. (XLSX)

S4 Dataset S1 Table noise: Extracted features of each manually labeled noise event. (XLSX)

## References

[1] Monge-Alvarez J, Hoyos-Barcelo C, Lesso P, Casaseca-de-la-Higuera P. Robust Detection of Audio-Cough Events Using Local Hu Moments. IEEE J Biomed Heal Informatics. 2019 Jan;23(1):184–96. doi: 10.1109/JBHI.2018.2800741 29994432

[2] Haji A, Kimura S, Ohi Y. A Model of the Central Regulatory System for Cough Reflex. Biol Pharm Bull. 2013;36(4):501–8. doi: 10.1248/bpb.b13-00052 23546286

[3] Magni C, Chellini E, Lavorini F, Fontana GA, Widdicombe J. Voluntary and reflex cough: Similarities and differences. Pulm Pharmacol Ther. 2011 Jun;24(3):308–11. doi: 10.1016/j.pupt.2011.01.007 21272659

[4] Aboalayon K, Faezipour M, Almuhammadi W, Moslehpour S. Sleep Stage Classification Using EEG Signal Analysis: A Comprehensive Survey and New Investigation. Entropy. 2016 Aug 23;18(9):272.

[5] Mendelson WB. Sleep Disorders Medicine. Fourth. Chokroverty S, editor. Sleep Disorders Medicine: Basic Science, Technical Considerations and Clinical Aspects. New York, NY: Springer New York; 2017. 5–28 p.

[6] Lee KK, Birring SS. Cough and Sleep. Lung. 2010 Jan 13;188(S1):91–4. doi: 10.1007/s00408-009-9176-0 19823913

[7] Landt EM, Çolak Y, Nordestgaard BG, Lange P, Dahl M. Risk and impact of chronic cough in obese individuals from the general population. Thorax. 2021 Jul 6;0:1–8. doi: 10.1136/thoraxjnl-2020-216351 34230095

[8] Gu W, Shangguan L, Yang Z, Liu Y. Sleep hunter: Towards fine grained sleep stage tracking with smartphones. IEEE Trans Mob Comput. 2016;15(6):1514–27.

[9] Zigel Y, Goldbart A, Freud T, Erew A, Abu Leil M, Tocker Y, et al. Diurnal and seasonal variation of cough episodes in healthy young adults. J Asthma. 2016 Mar 15;53(3):295–300. doi: 10.3109/02770903.2015.1087557 26513001

[10] Power JT, Stewart IC, Connaughton JJ, Brash HM, Shapiro CM, Flenley DC, et al. Nocturnal cough in patients with chronic bronchitis and emphysema. Am Rev Respir Dis. 1984;130(6):999–1001. doi: 10.1164/arrd.1984.130.6.999 6508020

[11] Sundar KM, Daly SE, Pearce MJ, Alward WT. Chronic cough and obstructive sleep apnea in a community-based pulmonary practice. Cough. 2010;6(1):1–7. doi: 10.1186/1745-9974-6-1 20398333 PMC2861010

[12] Sundar KM, Daly SE. Chronic Cough and OSA: An Underappreciated Relationship. Lung. 2014 Feb;192(1):21–5. doi: 10.1007/s00408-013-9534-9 24232979

[13] Sundar KM, Daly SE. Chronic Cough and OSA: A New Association? J Clin Sleep Med. 2011 Dec 15;07(06):669–77. doi: 10.5664/jcsm.1482 22171209 PMC3227716

[14] Chan K, Ing A, Birring SS. Cough in obstructive sleep apnoea. Pulm Pharmacol Ther. 2015 Dec;35:129–31. doi: 10.1016/j.pupt.2015.05.008 26068465

[15] Sundar KM, Willis AM, Smith S, Hu N, Kitt JP, Birring SS. A Randomized, Controlled, Pilot Study of CPAP for Patients with Chronic Cough and Obstructive Sleep Apnea. Lung. 2020 Jun 30;198(3):449–57. doi: 10.1007/s00408-020-00354-1 32356074 PMC8286636

[16] Franklin KA, Lindberg E. Obstructive sleep apnea is a common disorder in the population-A review on the epidemiology of sleep apnea. J Thorac Dis. 2015;7(8):1311–22. doi: 10.3978/j.issn.2072-1439.2015.06.11 26380759 PMC4561280

[17] Varga AW, Mokhlesi B. REM obstructive sleep apnea: risk for adverse health outcomes and novel treatments. Sleep Breath. 2019 Jun 19;23(2):413–23. doi: 10.1007/s11325-018-1727-2 30232681 PMC6424642

[18] Radine A, Werner C, Raidt J, Dougherty GW, Kerschke L, Omran H, et al. Comparison of Nocturnal Cough Analysis in Healthy Subjects and in Patients with Cystic Fibrosis and Primary Ciliary Dyskinesia: A Prospective Observational Study. Respiration. 2019;97(1):60–9. doi: 10.1159/000493323 30408808

[19] Zaibi H, Ben Jemia E, Kchok H, Dhahri B, Ben Amar J, Aouina H. Particularities of asthma in obese patients. Nutr Clin Métabolisme. 2021 Sep;35(3):207–11.

[20] Fontana GA, Widdicombe J. What is cough and what should be measured? Pulm Pharmacol Ther. 2007 Aug;20(4):307–12. doi: 10.1016/j.pupt.2006.11.009 17291801

[21] Ojoo JC, Everett CF, Mulrennan S a, Faruqi S, Kastelik J a, Morice AH. Management of patients with chronic cough using a clinical protocol: a prospective observational study. Cough. 2013;9(1):2. doi: 10.1186/1745-9974-9-2 23347748 PMC3565860

[22] Birring SS. Controversies in the evaluation and management of chronic cough. Am J Respir Crit Care Med. 2011;183(6):708–15. doi: 10.1164/rccm.201007-1017CI 21148722

[23] You M, Wang H, Liu Z, Chen C, Liu J, Xu X, et al. Novel feature extraction method for cough detection using NMF. IET Signal Process. 2017;11(5):515–20.

[24] Matos S, Birring SS, Pavord ID, Evans DH. Detection of Cough Signals in Continuous Audio Recordings Using Hidden Markov Models. IEEE Trans Biomed Eng. 2006 Jun;53(6):1078–83. doi: 10.1109/TBME.2006.873548 16761835

[25] Drugman T, Urbain J, Bauwens N, Chessini R, Aubriot AS, Lebecque P, et al. Audio and contact microphones for cough detection. In: INTERSPEECH 2012. 2012. p. 1302–5.

[26] Liu JM, You M, Wang Z, Li GZ, Xu X, Qiu Z. Cough event classification by pretrained deep neural network. BMC Med Inform Decis Mak. 2015;15(4):S2. doi: 10.1186/1472-6947-15-S4-S2 26606168 PMC4660085

[27] Amoh J, Odame K. Deep Neural Networks for Identifying Cough Sounds. IEEE Trans Biomed Circuits Syst. 2016 Oct;10(5):1003–11. doi: 10.1109/TBCAS.2016.2598794 27654978

[28] Simou N, Stefanakis N, Zervas P. A Universal System for Cough Detection in Domestic Acoustic Environments. In: 2020 28th European Signal Processing Conference (EUSIPCO). IEEE; 2021. p. 111–5.

[29] Miranda IDS, Diacon AH, Niesler TR. A Comparative Study of Features for Acoustic Cough Detection Using Deep Architectures *. In: 2019 41st Annual International Conference of the IEEE Engineering in Medicine and Biology Society (EMBC). IEEE; 2019. p. 2601–5. 10.1109/EMBC.2019.8856412 31946429

[30] Dafna E, Tarasiuk A, Zigel Y. Sleep staging using nocturnal sound analysis. Sci Rep. 2018 Dec 7;8(1):13474. doi: 10.1038/s41598-018-31748-0 30194402 PMC6128888

[31] Civera M, Filosi CM, Pugno NM, Silvestrini M, Surace C, Worden K. Assessment of vocal cord nodules: a case study in speech processing by using Hilbert-Huang Transform. J Phys Conf Ser. 2017 May;842:1–17.

[32] Markel J. The SIFT algorithm for fundamental frequency estimation. IEEE Trans Audio Electroacoust. 1972 Dec;20(5):367–77.

[33] Kalkbrenner C, Eichenlaub M, Brucher R. Development of a new homecare sleep monitor using body sounds and motion tracking. Curr Dir Biomed Eng. 2015 Sep 1;1(1):30–3.

[34] Li X, Li X, Zhang W, Liu Q, Gao Y, Chang R, et al. Factors and potential treatments of cough after pulmonary resection: A systematic review. Asian J Surg. 2021 Aug;44(8):1029–36. doi: 10.1016/j.asjsur.2021.01.001 33610443

[35] Pramono RXA, Imtiaz SA, Rodriguez-Villegas E. A Cough-Based Algorithm for Automatic Diagnosis of Pertussis. Hozbor DF, editor. One PLoS. 2016 Sep 1;11(9):e0162128. 10.1371/journal.pone.0162128 PMC5008773 27583523

[36] Fallahpour S, Lakvan EN, Zadeh MH. Using an ensemble classifier based on sequential floating forward selection for financial distress prediction problem. J Retail Consum Serv. 2017 Jan;34:159–67.

[37] Wang J, Xia B. Relationships of Cohen’s Kappa, Sensitivity, and Specificity for Unbiased Annotations. In: Proceedings of the 2019 4th International Conference on Biomedical Signal and Image Processing (ICBIP 2019). New York, New York, USA: ACM Press; 2019. p. 98–101.

[38] Perolat J, Couso I, Loquin K, Strauss O. Generalizing the Wilcoxon rank-sum test for interval data. Int J Approx Reason. 2015 Jan;56(PA):108–21.

[39] Ischaki E, Papatheodorou G, Gaki E, Papa I, Koulouris N, Loukides S. Body Mass and Fat-Free Mass Indices in COPD. Chest. 2007 Jul;132(1):164–9. doi: 10.1378/chest.06-2789 17505043

[40] Kelsall A, Decalmer S, McGuinness K, Woodcock A, Smith JA. Sex differences and predictors of objective cough frequency in chronic cough. Thorax. 2009 May 1;64(5):393–8. doi: 10.1136/thx.2008.106237 19131447

[41] Lai K, Huang L, Zhao H, Wu F, Zhen G, Deng H, et al. Clinical characteristics of patients with chronic cough in Guangdong, China: a multicenter descriptive study. BMC Pulm Med. 2021 Dec 27;21(1):305. doi: 10.1186/s12890-021-01642-z 34579688 PMC8477516

[42] Wimms A, Woehrle H, Ketheeswaran S, Ramanan D, Armitstead J. Obstructive Sleep Apnea in Women: Specific Issues and Interventions. Biomed Res Int. 2016;2016:1–9. doi: 10.1155/2016/1764837 27699167 PMC5028797

[43] Guilleminault L. Chronic cough and obesity. Pulm Pharmacol Ther. 2019 Apr 1;55:84–8. doi: 10.1016/j.pupt.2019.01.009 30817992

[44] Wang T-Y, Lo Y-L, Liu W-T, Lin S-M, Lin T-Y, Kuo C-H, et al. Chronic cough and obstructive sleep apnoea in a sleep laboratory-based pulmonary practice. Cough. 2013 Dec 5;9(1):24. doi: 10.1186/1745-9974-9-24 24188336 PMC4176501

